# The relationship of the efficiency of energy conversion into growth as an indicator for the determination of the optimal dose for mutation breeding with the appearance of chromosomal abnormalities and incomplete mitosis after gamma irradiation of kernels of *Triticum turgidum* ssp. *durum* L.

**DOI:** 10.1007/s00411-023-01026-3

**Published:** 2023-04-19

**Authors:** Eben Von Well, Annabel Fossey, Mardé Booyse

**Affiliations:** 1 ARC-Small Grain Institute, An Institute of the Field Crops Division, Private Bag X29, Bethlehem, 9700 South Africa; 2 Graduate Mastery, Boskruin View Office Park, 181 Girdwood Avenue, Bush Hill, Randburg, 2154 South Africa; 3 ARC Biometry, Private Bag X5013, Stellenbosch, 7599 South Africa

**Keywords:** Chromosomal abnormalities, Efficiency of energy conversion, Gamma irradiation, Incomplete mitosis, Micronuclei, Wheat

## Abstract

The study aim was to determine the optimal gamma irradiation dose for mutation breeding in *Triticum turgidum* ssp. *durum* L. Root, shoot and seedling growth, as well as the efficiency of energy conversion into growth were determined to examine the growth retardation effects of gamma irradiation that are the result of DNA damage (bridges, ring chromosomes, micronuclei, incomplete mitosis) in *Triticum turgidum* ssp. *durum* L. The kernels were irradiated with doses of 50, 150, 250 and 350 Gy using a ^60^Cobalt gamma-ray source. The kernels were placed in germination paper at 25 °C to grow for a 132 h period for the determination of shoot and root growth and the efficiency of energy conversion into growth. Root tips were collected and fixated over a 47.5 h growth period for the determination of the chromosomal abnormalities and incomplete mitosis. The control differed highly significantly (*p* < 0.01) from irradiated samples at all doses in root growth and from 250 to 350 Gy samples in shoot growth and the efficiency of energy conversion into growth. There was a highly significant (*p* < 0.01) increase in the number of bridges and micronuclei between 50 Gy samples and samples irradiated with the higher irradiation doses while 50 Gy samples differed only from 250 and 350 Gy samples regarding ring chromosomes and interphase cells with incomplete mitosis. Root and seedling growth on the one hand and the efficiency of energy conversion into growth on the other were found to be measuring different effects of gamma irradiation on plant growth. The latter was used for the determination of the optimal dose for mutation breeding as 155.52 Gy.

## Introduction

Mutation breeding in plants is used to generate new variations that will lead to better adaptation to biotic and abiotic stresses. Treatment with chemical or irradiation mutagens is used to generate the variations. Gamma irradiation of seeds is the most widely used form of irradiation treatment. For the gamma irradiation to be effective, it is necessary to determine the dose that will generate the largest amount of variation in the M_2_ generation (i.e., second generation after irradiation). There are two methods used to predict the ideal dose for mutation breeding. Firstly, a 50% growth reduction of seedling height (GR_50_) after acute gamma irradiation of dormant kernels is widely used as a measure of irradiation damage to obtain the ideal irradiation dose for mutation breeding. It is used because it is easy to predict the GR_50_. In cereals, measurements are taken when the shoots of the control seedlings are between 11 and 20 cm, usually after 10 to 14 days, for plants in sowing trays. Seedling height is measured from the soil level to the tip of the primary leaf in cereals. It became clear in recent years that the GR_50_ is not sensitive enough to predict the ideal gamma irradiation dose for mutation breeding and it predicts a value that is higher than ideal. As an example, the GR_50_ range for *Triticum turgidum* ssp. *durum* L. is 350–500 Gy, while the ideal gamma irradiation dose range for mutation breeding is 150–300 Gy (FAO/IAEA 2018). Secondly, determination of the maximum mutation rate, *M*_max_, is a different viewpoint for the prediction of the ideal dose for mutation breeding by using the following equation (Ukai 2006):

1 $$M_{{{\text{max}}}} = k_{{\text{m}}} D_{{{\text{max}}}}$$

where *M*_max_ is the product of mutation rate per unit dose (*k*_m_) and the maximum dose applicable (*D*_max_).

M_1_ trades that are measured for the determination of *M*_max_ are: germination rate, seedling growth, root growth, survival rate, number of spikes and seed fertility. The higher the mutagen dose applied, the more severe the injuries become. Lethal effects prevent raising of the dose of irradiation beyond a certain threshold value of *D*_max_ (Ukai 2006).

The two methods used for the determination of the ideal dose for mutation breeding make use of growth reduction. It is therefore important to examine the main factors that are playing a role in growth reduction. Physiological processes, DNA repair, as well as cell death (Lagoda 2012) influence this reduction in growth. Physiological processes may lead to increased growth at low dose levels and decreased growth at higher dose levels (Borzouei et al. 2010). Gamma irradiation damages and breaks chromatin in the G1, S and G2 cell stages during kernel treatment (Hase et al. 1999). It has been reported by several authors that the percentage of abnormal cells increases as the irradiation dose increases (Viccini and de Carvalho 2002). Various ratios of irradiation-induced aberrations have been used as possible fingerprints of irradiation quality (Ballarini and Ottolenghi 2003; Liu et al. 2013).

The effects of gamma irradiation on growth parameters differ in the *Triticum* polyploid complex. In diploid wheat, *Triticum monococcum* L. (2n = 2x = AA = 14) a 50 Gy gamma irradiation dose resulted in a significant reduction in shoot and root growth and mobilisation of food reserves (Von Well et al. 2017). The *Triticum* polyploid complex also consists of tetraploid (2n = 4x = AABB = 28) and hexaploid (2n = 6x = AABBDD = 42) species (Dvorak 2013). Gamma irradiation dose levels that lead to a decrease in growth and physiological processes in diploid wheat, can lead to an increase in the polyploid species. In *Triticum aestivum* L. low gamma irradiation doses (10–100 Gy) resulted in improved flag leave area and photosynthetic traits, as well as a higher nutrient uptake of the roots (Singh and Datta 2010a). Considering the irradiation dose related uniformity in crop performance, the plant response appears to have been realized at the physiological rather than genetic level in polyploid wheat (Singh and Datta 2010b). Increases in Chlorophyll and proline content, as well as shoot and root dry weights after 100 Gy gamma irradiation of seed of hexaploid wheat were found to differ in different genotypes (Borzouei et al. 2010). An increase in root number and length occurred in hard wheat (*Triticum turgidum* ssp. *durum* L.) after irradiation with relatively low dose (20 Gy) of the seeds (Melki and Dahmani 2009; Melki and Marouani 2010). Gamma irradiation with 300 Gy (high dose) significantly reduced germination, survival percentage and plant height, while significantly increasing the time to spike initiation and maturation. Furthermore, fourteen different chlorophyll mutations at various frequencies were obtained compared to M_0_ plants for a *Triticum turgidum* ssp. *durum* L. variety (Ahumada-Flores et al. 2021).

Gamma irradiation effects on chromosomal level, which can lead to physiological effects as well as cell death, are observed by means of cytogenetic analysis. The cytokinesis-block micronucleus (CBMN) assay is now commonly used to determine irradiation sensitivity of tumour cells and cytotoxicity in animal and human cell cultures (Fenech 2008; Mendes et al. 2019). The CBMN assay can, however, not be used for the determination of mitotic indexes and the distribution of micronuclei in daughter cells. The following abnormalities can be observed during mitosis in plants after X-ray and gamma irradiation: stickiness and clumping of chromosomes, diplochromosomes or pseudochiasma, rings, fragments, bridges with or without fragments, micronuclei, giant cells, cellular and nuclear shape deformities, disrupted equatorial plates and uncoiling chromosomes at metaphase (Gupta et al. 2019; Oney-Birol and Balkan 2019). Over time micronuclei can be extruded from the cell, reincorporated in the nucleus, be degraded or persist in the cytoplasm of the cell. Ring chromosomes are also carried over separately from one cell cycle to another (Geard 1976). These micronucleated cells may survive several cycles of mitosis or are eliminated by means of apoptosis (Hintzsche et al. 2017).

We created the “efficiency of energy conversion into growth” as a predictor of the ideal dose for mutation breeding to replace seedling height (GR_50_) and the *M*_max_ determination (Von Well et al. 2017). It makes use of a reduction in growth as well as the amount of energy (reserve food in the caryopsis) used to obtain that growth. Respiration rate, calculated as the difference between the initial kernel dry weight and the actual dry weights of the shoot, roots and caryopsis during a specific period of growth (Bouaziz and Hicks 1990), is normally used to predict inefficiency of energy usage for growth in plants due to an external factor. Respiration cannot be used to predict the ideal dose for mutation breeding, because it is dependent on the metabolic activity of the plant that decreases with an increase in irradiation dose (Von Well et al. 2017). The efficiency of energy conversion into growth is calculated as:

2 $${ = }\frac{{\left( {\text{combined shoot and root dry weight}} \right) - \left( {\text{original embryo dry weight}} \right)}}{{\left( {\text{original caryopsis dry weight}} \right) - \left( {\text{actual dry weight of caryopsis at a particular point of time}} \right)}}$$

By dividing the actual growth rate by the energy used to obtain the specific growth rate, the metabolic activity does not have a decreasing effect on the value of the inefficiency of energy conversion into growth with an increase in gamma irradiation dose. The fact that the whole plant (shoot and root dry weight) is used, makes it more sensitive to gamma irradiation than if only the shoot dry weight had been used, because the root growth is more sensitive than shoot growth (Von Well et al. 2017). In *Triticum monococcum* L., the ideal gamma irradiation dose range for mutation breeding is 100–values of pairwise comparisons of gamma irradiation dose for 200 Gy (FAO/IAEA 2018). By making use of the efficiency of energy conversion into growth, 100 Gy was predicted to be the ideal dose for mutation breeding in Einkorn (Von Well et al. 2017).

The retarding effect on shoot and root growth, efficiency of energy conversion into growth, as well as cytogenetic effects of gamma irradiation were studied in tetraploid *Triticum turgidum* ssp. *durum* L. The first aim was to determine whether root and seedling growth and the efficiency of energy conversion into growth are measuring different physiological processes that result in growth retardation as measured by bridges, ring chromosomes, micronuclei and incomplete mitosis. The second aim was to determine the ideal dose for mutation breeding by using the efficiency of energy conversion into growth, if found to be measuring different physiological processes. Complete repair of meristematic cells only takes place until a certain degree of damage is obtained by the cells. When the damage to the meristematic cells is above a threshold value, incomplete repair takes place. These damaged cells stop dividing and may even be eliminated. Full repair of meristematic cells leads to full recovery and effective energy conversion into growth. However, as the percentage of repaired meristematic cells that participate in the mitotic cell divisions decreases, so does the efficiency of recovery and thus energy conversion into growth decreases (Von Well et al. 2017). It is expected, therefore, that root and seedling growth will have a better correlation with a low number of bridges/micronuclei per cell, while the efficiency of energy conversion into growth will have a better correlation with many bridges/micronuclei and ring chromosomes per cell.

## Materials and methods

### Kernel preparation for gamma irradiation and imbibition

Kernels of *Triticum turgidum* ssp. *durum* L. cv. Orania were obtained from the Small Grain Germplasm Collection at Agricultural Research Council–Small Grain Institute.

Kernel size affects early growth and seedling vigour (Hampton 1981; Lafond and Baker 1986). The kernels used for this investigation were therefor selected to weigh within 2 mg of the average kernel weight of 46 mg. To ensure repeatability of the experiments, the moisture content of the kernels was adjusted to 14% prior to irradiation (FAO/IAEA 2018). The kernels were given gamma irradiation doses of 50, 150, 250 and 350 Gy using a ^60^Cobalt source at the South African National Blood Services in Roodepoort, Gauteng, South Africa. These irradiation doses were used to produce an increase in metabolic activity and growth rate at 50 Gy, while the higher irradiation doses are expected to lead to a decrease in metabolic activity and growth rate. The lower growth rate may also be due to cell cycle arrest in G2 phase, unsuccessful cell division at the highest doses and cell death, as observed in irradiation of cancer cells (Xu et al. 2002; Nazarenko and Kharytonov 2016).

The imbibition conditions for the experiments were as follows: the gamma irradiated kernels, as well as kernels that were not irradiated, which acted as the control, were placed between standard germination papers in an incubator in the dark at a constant temperature of 25 °C, according to the regulations of ISTA (ISTA 2015).

### Shoot and root growth and determination of the efficiency of energy conversion into growth

The shoot and root growth, as well as the efficiency of energy conversion into growth were assessed from 60 h after imbibition when the primary roots and shoots had emerged, reserve food transportation from the caryopsis to the embryo had commenced and active growth is taking place in unirradiated and irradiated material. The growth period that ended after 132 h of imbibition was used to give samples after the lower gamma irradiation doses time to recover and after the higher doses time to deteriorate. The 132 h was long enough for the efficiency of energy conversion into growth to recover at 50 Gy treatment in *Triticum monococcum* L. (Von Well et al. 2017). The growth rate of *Triticum turgidum* ssp. *durum* L. is higher than that of the diploid (Von Well and Fossey 2002), therefore, 132 h was chosen to be the ideal growth period for recovery or deterioration of the efficiency of energy conversion into growth in the *Triticum* polyploid complex. Caryopses and seedlings were collected every 12 h, eight kernels at a time, starting at 60 h after the initiation of imbibition. Caryopses and seedlings were then dried for at least 48 h in an oven at 105 °C, after which the dry weights of the shoot, root and remaining caryopsis, were recorded.

Shoot and root growth were determined by the increase in shoot and root dry weights (Bouaziz and Hicks 1990).

The initial embryo dry weight was recorded by drying eight embryos and determination of the average dry weight of the embryos.

The initial caryopsis dry weight of all the caryopses collected at specific 12 h intervals required the determination of a regression equation for kernel weight at 14% moisture content and caryopsis dry weights before the onset of imbibition. First, eight kernels were weighed. Then, the caryopses were separated from the embryos. Following that, the caryopses were dried for at least 48 h in an oven at 105 °C, after which the dry weight measurements were recorded. After that, a regression equation was fitted to the initial kernel weight at 14% moisture content and caryopsis dry weights. Finally, the initial dry weight of the caryopsis was calculated according to the following equation:

3 $$y \, = {\text{ a }} + {\text{ bx}}$$

4 $$y \, = \, - {6}.{46 } + { 1}.0{\text{3x}}$$

where *y* = the predicted initial dry weight of the caryopsis, *a* = regression line intercept, *b* = regression line slope, *x* = initial kernel weight at 14% moisture content

The efficiency of energy conversion into growth was calculated by making use of the Eq. 2 in the Introduction (Von Well et al. 2017).

### Fixation and slide preparation for analysis of bridges, ring chromosomes and micronuclei

The fixation and slide preparations were according to the combination of two methods (Johansen 1940; Pienaar 1955). Five roots were collected every two and a half hours from 17.5–47.5 h after onset of imbibition for each treatment and fixed in La Cour’s 2BD for 8 h. The roots were then bleached in a 1:20 mixture of saturated aqueous solution of ammonium oxalate and (6%) hydrogen peroxide for different time periods for each dose (Table 1). For the squash preparations, the roots were first macerated in 15% pectinase for 20 h at 30 °C and then hydrolysed in 1N HCl at 60 °C for 15 min (non-irradiated, 50 Gy and 150 Gy irradiation) or 12 min (250 Gy and 350 Gy irradiation) and stained in Feulgen (Pienaar 1955) for two and a half hours. After a 10-min rinse, squash preparations were prepared by squashing the meristematic root tip (first 1 mm) in 45% acetic acid on a slide. An albumenised coverslip was placed on the material, followed by gentle tapping and squashing between two layers of filter paper. The coverslip was dehydrated by passing it through 70% ethanol and 96% ethanol for two minutes in each solution. The coverslips were then counter stained in 0.25% Fast Green (Johansen 1940) for 5 min and processed in 100% ethanol for two minutes. The ethanol was removed by placing the coverslip in 100% ethanol:xylene (1:1) for three minutes and then in xylene for a further 3 min and finally mounted with Entellan.

**Table 1:** Bleaching times for the different gamma irradiation doses

Dose (Gy)	Length of bleaching time (min)
0	15.0
50	14.5
150	13.0
250	11.5
350	9.5

### Cytogenetic analysis

The effect of gamma irradiation on the appearance of bridges, ring chromosomes, micronuclei and incomplete mitosis in meristematic cells were assessed. The determination of the number of bridges was done by scoring the number of bridges in anaphase and telophase cells out of 500 meristematic cells. The number of ring chromosomes were determined by scoring the number of rings in prophase to anaphase cells out of 500 meristematic cells. The number of micronuclei in interphase cells were determined by scoring 500 interphase cells. The number of incomplete mitosis were determined by scoring the number of interphase cells with five and more nucleoli in the nucleus out of 500 interphase cells (Von Well et al. 2021). The mean number of bridges, ring chromosomes and micronuclei per 500 cells and the frequency of cells with different numbers of bridges, ring chromosomes and micronuclei were determined for the evaluations.

### Experimental layout and statistical analysis

The experimental layout for shoot and root growth evaluation and evaluation of the efficiency of energy conversion into growth was a randomized complete block design with eight replicates (blocks). A two-factor factorial experimental design was followed with three dependent variables with dose levels of 0, 50, 150, 250 and 350 Gy and time with levels 60, 72, 84, 96, 108, 120 and 132 h. The effects of the factors dose and time and dose by time interactions were tested using the GLM procedure of SAS software (SAS Institute 2012 North Carolina 27,513: SAS Campus Drive, Cary). The least significant means (LSMEANS) procedure of SAS was used to compare the effects of the different gamma irradiation doses on shoot and root growth and efficiency of energy conversion into growth. The average shoot and root growth and efficiency of energy conversion into growth per gamma irradiation dose were displayed in tables and the effects of the gamma irradiation were expressed as *p* values, where *p* = 0.05 resembled a significant effect and *p* < 0.01 resembled a highly significant effect.

The experimental layout for bridges, ring chromosomes, micronuclei and incomplete mitosis evaluations was a randomized complete block design with five replicates (blocks). A two-factor factorial experimental design was followed with three dependent variables with dose levels of 0, 50, 150, 250 and 350 Gy and time levels 17.5, 20, 22.5, 25, 27.5, 30, 32.5, 35, 37.5, 40, 42.5, 45 and 47.5 h. The effects of the factors dose and time and dose by time interactions were tested using the GLM procedure of SAS software (SAS Institute 2012 North Carolina 27,513: SAS Campus Drive, Cary). The least significant means (LSMEANS) procedure of SAS was used to compare the effect of the different gamma irradiation doses on the presence of bridges, ring chromosomes, micronuclei and incomplete mitosis. The average number of bridges, ring chromosomes, micronuclei and incomplete mitosis per gamma irradiation dose were displayed in tables and the effects of the gamma irradiation were expressed as *p* values, where *p* = 0.05 resembled a significant effect and *p *< 0.01 resembled a highly significant effect.

### Correlations

Pearson’s correlation coefficients were determined between root and seedling growth as well as the efficiency of energy conversion into growth on the one hand and the numbers of cells with different numbers of micronuclei/bridges and incomplete mitosis on the other. Root growth was used for the correlations because the cytogenetic study was done on root meristematic cells. Seedling growth was used because seedling growth and not root growth was used for the determination of efficiency of energy conversion into growth and to see if there is a large difference between the correlations using root dry weight or seedling dry weight. Root/seedling growth represent retardation in growth due to cell death as well as decreased metabolic/physiologic activity. The efficiency of energy conversion into growth decreases due to cell death and energy used for repairing of damaged cells. The numbers of cells with different numbers of micronuclei/bridges and incomplete mitosis were the abnormalities observed in the cytogenetic study and therefor used to explain what happens during root and shoot growth retardation and decrease in efficiency of energy conversion into growth.

### Determination of the optimum dose for mutation breeding

The efficiency of energy conversion into growth will be used to determine the ideal dose for mutation breeding if it is found to be measuring different aspects of growth retardation than root growth and seedling growth. In *Triticum monococcum* L. the 100 Gy gamma irradiation treatment was not entangled with the control and differed from the control according to LSMEANS procedure with a *p* = 0.01 value (Von Well et al. 2017). The 100 Gy gamma irradiation dose predicted as the optimal dose for mutation breeding falls in the range as prescribed by the FAO/IAEA (FAO/IAEA 2018) for *Triticum monococcum* L. The *p* = 0.01 value may be used as a guide for predicting the optimal dose for mutation breeding.

## Results

The effect of gamma irradiation on tetraploid wheat, *Triticum turgidum* ssp. *durum* L. cv. Orania, was investigated by studying the effect on growth parameters such as shoot and root growth and the efficiency of energy conversion into growth and the effect on chromatin in the form of chromatin bridges (break in one chromosome arm), ring chromosomes (breaks in both chromosome arms), micronuclei and incomplete mitosis.

### Shoot growth rates

**Fig. 1:**
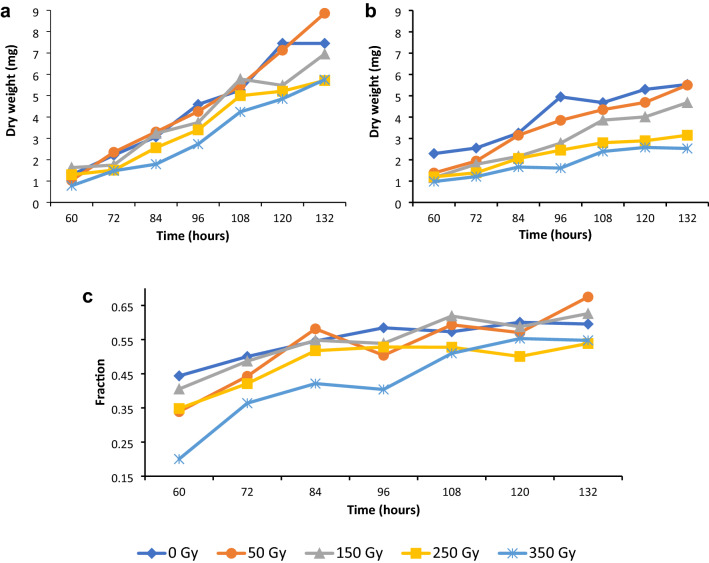
Effect of gamma irradiation doses of 0, 50, 150, 250 and 350 Gy on **a** shoot growth means, **b** root growth means, **c** efficiency of energy conversion into growth fractions in *T. turgidum* ssp. *durum* L. cv. Orania

Shoot growth rate was assessed over time to investigate metabolic activity as affected by the gamma irradiation. Shoot growth of the irradiated seed displayed growth retardation when compared to the control group, especially at higher doses (Fig. 1a). Shoot growth by means of the ANOVA tests revealed highly significant effects of dose, as well as dose by time interactions (Table 2). Shoot growth analysis according to the LSMEANS procedure showed highly significant differences for all pairs of shoot growth, except for the treatment pairs of control and 50 Gy, control and 150 Gy, as well as 50 Gy and 150 Gy, which were non-significant (Table 3).

**Table 2:** Two-way ANOVA showing sources of variation for shoot and root growth and efficiency of energy conversion into growth in *T. turgidum* ssp. *durum* L. cv. Orania

Source	DF	Type I SS	Mean square	*F* value	Pr > *F*
Shoot growth					
Block	7	6.831	0.976	1.33	0.24
Dose	4	93.773	23.443	31.83	< 0.01
Time	6	1120.643	186.774	253.62	< 0.01
Dose × time	24	55.021	2.293	3.11	< 0.01
Root growth					
Block	7	4.134	0.591	1.27	0.64
Dose	4	184.386	46.096	98.98	< 0.01
Time	6	185.668	47.611	102.24	< 0.01
Dose × time	24	37.089	1.545	3.32	< 0.01
Efficiency of energy conversion into growth					
Block	7	0.043	0.006	1.08	0.377
Dose	4	0.582	0.146	25.72	< 0.01
Time	6	1.772	0.295	52.19	< 0.01
Dose × time	24	0.268	0.011	1.97	< 0.01

**Table 3:** *p* values of pairwise comparisons of gamma irradiation dose for shoot and root growth and efficiency of energy conversion into growth in *T. turgidum* ssp. *durum* L. cv. Orania

*p* values of least squares means comparison										
*T. turgidum* ssp. *durum* L. cv. Orania										
Shoot growth (mg)						Root growth (mg)				
Average	**4.47**	**4.63**	**4.08**	**3.53**	**3.09**	**4.08**	**3.55**	**2.92**	**2.28**	**1.85**
Dose (Gy)	**0**	**50**	**150**	**250**	**350**	**0**	**50**	**150**	**250**	**350**
0		**0.34**	0.02	< 0.01	< 0.01		< 0.01	< 0.01	< 0.01	< 0.01
50			< 0.01	< 0.01	< 0.01			< 0.01	< 0.01	< 0.01
150				< 0.01	< 0.01				< 0.01	< 0.01
250					0.01					< 0.01

Efficiency of energy conversion into growth										
Average	**0.549**	**0.529**	**0.545**	**0.483**	**0.429**					
0		**0.17**	**0.75**	< 0.01	< 0.01					
50			**0.29**	< 0.01	< 0.01					
150				< 0.01	< 0.01					
250					< 0.01					

Non-significant differences in bold *p* = 0.05 significant effect and *p* < 0.01 highly significant effect

### Root growth rates

Also root growth rate was assessed over time to investigate metabolic activity after gamma irradiation. Root growth of the irradiated seed also displayed growth retardation when compared to the control group. Retardation showed an increase with increasing irradiation doses (Fig. 1b). Root growth by means of the ANOVA tests revealed highly significant effects of time and dose, as well as dose by time interactions (Table 2). Root growth analysis according to the LSMEANS procedure showed highly significant differences for all pairs of root growth (Table 3).

### Efficiency of energy conversion into growth

The efficiency of energy conversion over time was investigated to observe the effect of gamma irradiation on energy spending for growth and repairing of damage caused by the gamma irradiation that changes over time (Fig. 1c). Efficiency of energy conversion into growth by means of the ANOVA tests showed highly significant effects of dose and dose by time interactions. The time effect was slightly significant (Table 2). Efficiency of energy conversion into growth analysis according to the LSMEANS procedure revealed that most treatment pairs demonstrated highly significantly differences in efficiency of energy conversion into growth except for the following treatment pairs: control with 50 Gy, control with 150 Gy, as well as 50 Gy with 150 Gy (Table 3).

### Presence of bridges

**Fig. 2:**
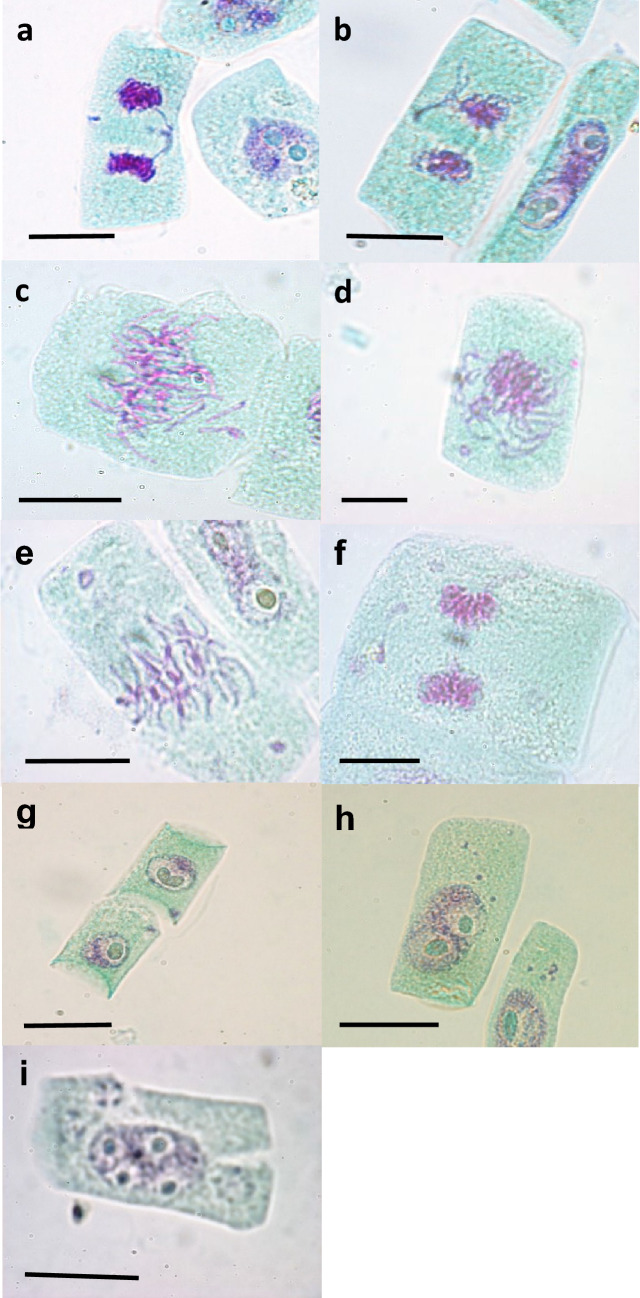
Chromosomal abnormalities in *T. turgidum* ssp. *durum* L. cv. Orania. **a** A side-arm bridge due to half chromatid breakage. **b** Early telophase with one intact bridge and two broken bridges. **c** Metaphase with three fragments. **d** Late prophase with two fragments and a ring chromosome. **E** Metaphase with two ring chromosomes of different sizes. **f** Dividing ring chromosome in late anaphase. **g** Two daughter cells, each containing a micronucleus. **h** Interphase cell containing three micronuclei. **i** Five nucleoli in the nucleus as well as small nucleoli in micronuclei in interphase cell with incomplete mitosis. Bar = 10 µm

**Fig. 3:**
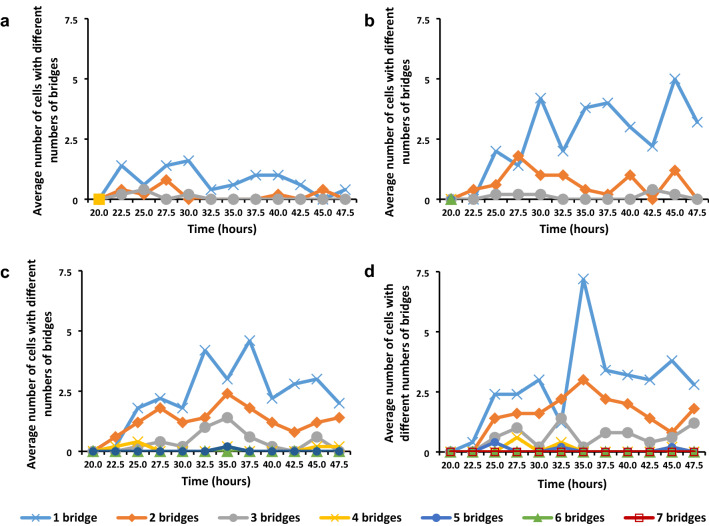
Average number of cells (per 500 cells) with different numbers of bridges at **a** 50 Gy, **b** 150 Gy, **c** 250 Gy and **d** 350 Gy in *T. turgidum* ssp. *durum* L. cv. Orania

The presence of bridges in anaphase (Fig. 2a) and telophase (Fig. 2b) was assessed over the period from 17.5 to 47.5 h after the onset of imbibition to visualise the effect of the gamma irradiation on the chromosomes during mitosis (Fig. 3). In the investigation it was not always possible to determine if a bridge was single stranded or double stranded, therefore, all bridges were assessed together without this distinction. However, evidence of the presence of chromatin breaks due to gamma irradiation first became evident in the form of bridges at 22.5 h. The number of cells containing different numbers of bridges increased with an increase in irradiation dose. Samples treated with 50 Gy and 150 Gy displayed cells with one to three bridges. The number of cells with more than one bridge became rarer with an increase in number of bridges. Cells with two and three bridges occurred rarely in 50 Gy treated material, while 150 Gy treated material had marginally more of these cells with a substantial increase in cells with a single bridge. 250 Gy and 350 Gy treated material displayed many more cells with chromatin bridges, ranging from cells with one, to cells with seven bridges. Broken bridges were visible in telophase (Fig. 2b) and newly divided cells. Fragments were visible in metaphase (Fig. 2c) and anaphase.

**Fig. 4:**
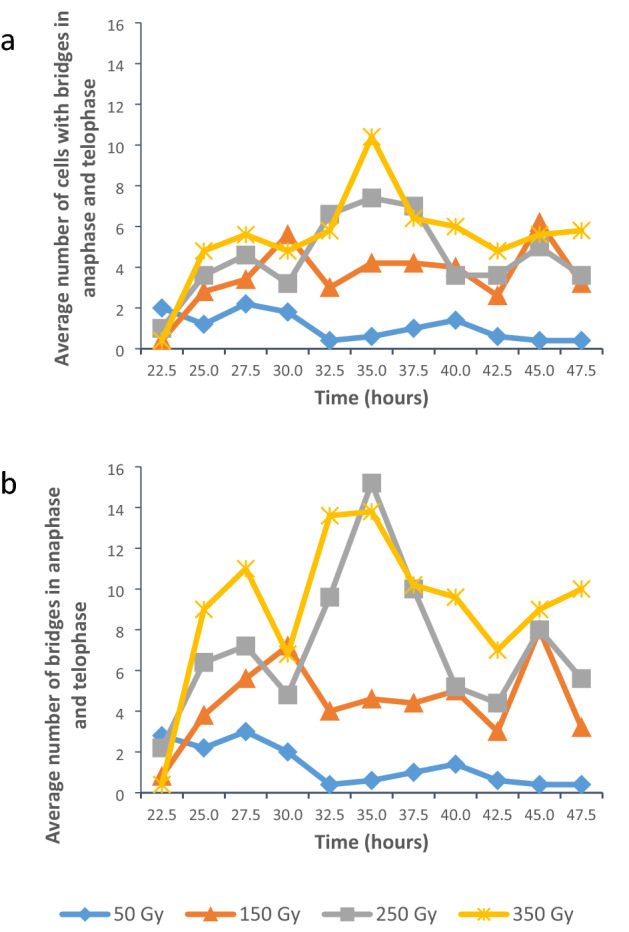
Bridges in anaphase and telophase cells in *T. turgidum* ssp. *durum* L. cv. Orania. **a** Average number of cells (per 500 cells) with bridges in anaphase and telophase. **b** Average number of bridges in anaphase and telophase (in 500 cells)

The average number of anaphase and telophase cells containing bridges clearly showed an increase with an increase in gamma irradiation dose (Fig. 4a). Throughout the assessment period the number of cells displayed periods of an increase and periods with a decrease in the number of cells containing bridges at all doses.

The extent of DNA damage through gamma irradiation was also assessed by determining the average number of bridges over the investigating period (Fig. 4b). There were large similarities between the average number of anaphase and telophase cells containing bridges and average number of bridges. The similarities decreased with an increase in gamma irradiation. The average number of bridges by means of the ANOVA tests showed highly significant effects of dose, time and dose by time interaction (Table 4).

**Table 4:** Two-way ANOVA showing sources of variation for total number of bridges, total number of ring chromosomes, total number of micronuclei and incomplete mitosis in *T. turgidum* ssp. *durum* L. cv. Orania

Source	DF	Type I SS	Mean square	*F* value	Pr > *F*
Bridges					
Block	4	10.785	2.696	0.26	0.90
Dose	4	3222.095	805.524	77.41	< 0.01
Time	10	536.502	53.650	5.16	< 0.01
Dose × time	40	998.225	24.956	2.40	< 0.01
Ring chromosomes					
Block	4	2.996	0.749	1.31	0.27
Dose	4	42.524	10.631	18.61	< 0.01
Time	10	50.102	5.010	8.77	< 0.01
Dose × time	40	69.716	1.743	3.05	< 0.01
Micronuclei					
Block	4	1137.287	284.322	0.21	0.94
Dose	4	3,713,260.887	928,315.222	672.20	< 0.01
Time	10	1,209,263.287	120,926.329	87.56	< 0.01
Dose × time	40	1,907,249.513	47,681.238	34.53	< 0.01
Incomplete mitosis					
Block	4	2.776	0.694	1.13	0.34
Dose	4	108.816	27.204	44.28	< 0.01
Time	9	27.636	3.071	5.00	< 0.01
Dose × time	36	75.664	2.102	3.42	< 0.01

The effect of gamma irradiation on the average number of bridges according to the LSMEANS procedure revealed that the differences among all irradiation treatments were highly significantly different (Table 5.).

**Table 5:** *p* values of pairwise comparisons of gamma irradiation dose for average number of bridges, ring chromosomes, micronuclei and interphase cells with incomplete mitosis in *T. turgidum* ssp. *durum* L. cv. Orania

*p* values of least squares means comparison										
*T. turgidum *ssp. *durum *L. cv. Orania										
Average number of bridges						Average number of ring chromosomes				
Average	**0.00**	**1.33**	**4.47**	**7.15**	**9.13**	**0.00**	**0.16**	**0.53**	**0.75**	**1.09**
Dose (Gy)	**0**	**50**	**150**	**250**	**350**	**0**	**50**	**150**	**250**	**350**
0		0.03	< 0.01	< 0.01	< 0.01		**0.26**	< 0.01	< 0.01	< 0.01
50			< 0.01	< 0.01	< 0.01			0.01	< 0.01	< 0.01
150				< 0.01	< 0.01				**0.13**	< 0.01
250					< 0.01					0.01

Average number of micronuclei						Average number of interphase cells with incomplete mitosis				
Average	**0.00**	**26.25**	**109.36**	**200.36**	**315.67**	**0.00**	**0.12**	**0.24**	**1.22**	**1.64**
0		< 0.01	< 0.01	< 0.01	< 0.01		**0.44**	**0.13**	< 0.01	< 0.01
50			< 0.01	< 0.01	< 0.01			**0.44**	< 0.01	< 0.01
150				< 0.01	< 0.01				< 0.01	< 0.01
250					< 0.01					0.01

Non-significant differences in bold *p* = 0.05 significant effect and *p* < 0.01 highly significant effect

### Presence of ring chromosomes

Ring chromosomes result from chromosomal breaks in both chromosome arms, followed by the fusion of the sticky ends to form a ring chromosome. Ring chromosomes were observable from late prophase (Fig. 2d), through metaphase (Fig. 2e) and in anaphase (Fig. 2f). A maximum of two ring chromosomes were observed per cell. The ring chromosomes divide at a later stage in anaphase than the rest of the chromosomes (Fig. 2f).

**Fig. 5:**
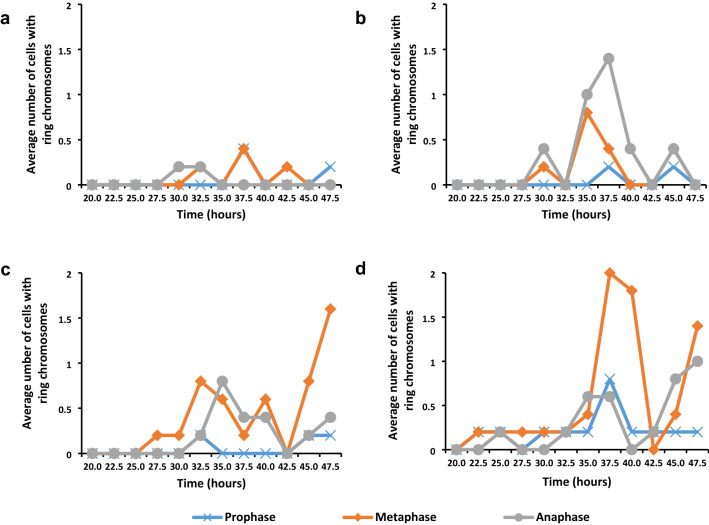
Average number of prophase, metaphase and anaphase cells (per 500 cells) with ring chromosomes at **a** 50 Gy, **b** 150 Gy, **c** 250 Gy and **d** 350 Gy in *T. turgidum* ssp. *durum* L. cv. Orania

An investigation of the presence of ring chromosomes in dividing cells revealed that, in general, ring chromosomes appeared earlier in prophase with an increase of gamma irradiation dose, however, the exception was 50 Gy material, that did reveal ring chromosomes earlier than 150 Gy material (Fig. 5). First appearance of ring chromosomes in metaphase and anaphase followed the same pattern as for prophase, except for 50 Gy material in which ring chromosomes in metaphase and anaphase were observed later than first appearance in prophase. The mean number of ring chromosomes showed a steady increase with an increase in dose. Most of the ring chromosomes were observed in metaphase cells.

**Fig. 6:**
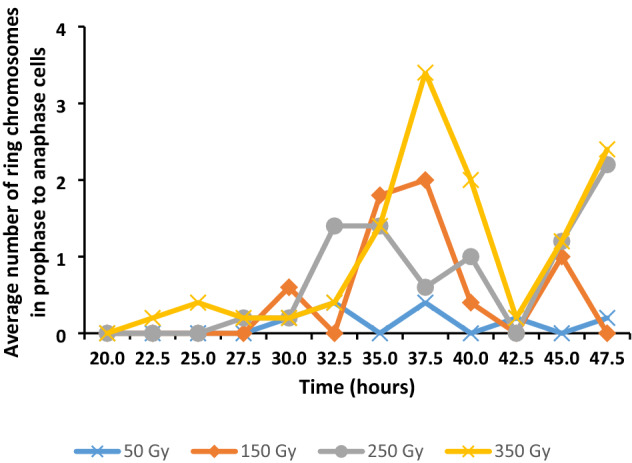
Average number of ring chromosomes in prophase, metaphase and anaphase cells at different gamma irradiation doses in *T. turgidum* ssp. *durum* L. cv. Orania

A comparison of the average number of ring chromosomes revealed the average number of ring chromosomes increased with an increase in gamma irradiation dose (Fig. 6). Fifty Gy material displayed the least number of cells with ring chromosomes and 350 Gy material the greatest number, while 150 Gy and 250 Gy material displayed an intermediate number of cells with ring chromosomes. The presence of ring chromosomes displayed a cyclic pattern with little or no cells with ring chromosomes to peaks of cells with ring chromosomes. The highest peak in 50 Gy material had an average of 0.4 ring chromosomes and the highest peak in 350 Gy material had an average of 3.4 ring chromosomes. It appeared that the number of peaks decreased with an increase of irradiation dose. 50 Gy and 150 Gy each had three peaks, while 250 Gy and 350 Gy each had two peaks.

The extent of DNA damage through gamma irradiation was also assessed by determining the total number of ring chromosomes over the investigating period. The total number of ring chromosomes by means of the ANOVA tests showed highly significant effects of dose, time and dose by time interaction (Table 4).

The effect of gamma irradiation on the total number of ring chromosomes according to the LSMEANS procedure revealed moderate to highly significant differences among the irradiation dose treatments except between the control and 50 Gy and between 150 and 250 Gy treatments that were non-significant (Table 5.).

### Presence of micronuclei

Acentric fragments become micronuclei towards the end of telophase when nuclear membranes are formed. Once fragments are formed into micronuclei, they are passed to daughter cells (Fig. 2g, h) by chance. They become visible at telophase and are present until prophase. During late prophase, metaphase and anaphase they lose their membranes and are visible as acentric fragments. In this investigation, the greatest number of micronuclei recorded per cell at interphase was seven.

**Fig. 7:**
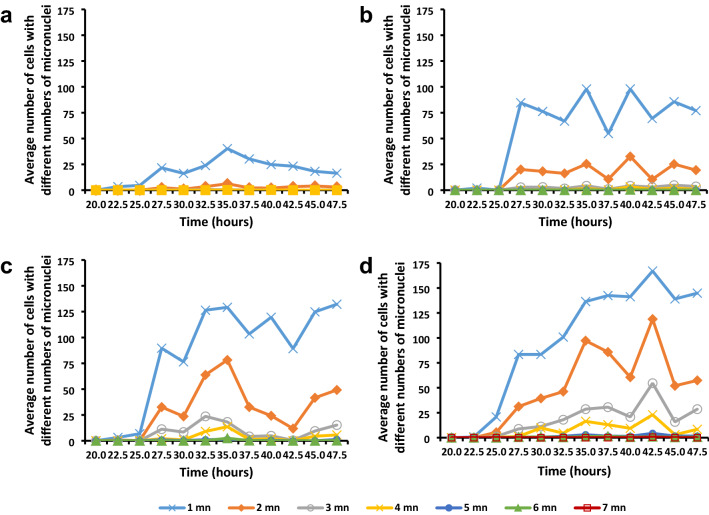
Average number of cells (per 500 cells) with different numbers of micronuclei at different irradiation doses. **a** 50 Gy, **b** 150 Gy, **c** 250 Gy and **d** 350 Gy in *T. turgidum* ssp. *durum* L. cv. Orania

The range and numbers of cells containing different numbers of micronuclei increased with an increase in gamma irradiation dose (Fig. 7). Fifty Gy material displayed only cells with one or two micronuclei; 150 Gy material displayed cells with one to four micronuclei, while in 250 Gy and 350 Gy material the number of micronuclei ranged from one to seven. In all the treatments, cells with one micronucleus were most frequently, followed by cells with two micronuclei. Cells with three and four micronuclei occurred at lower frequencies, while cells with five to seven micronuclei occurred rarely, less than five cells out of 500.

**Fig. 8:**
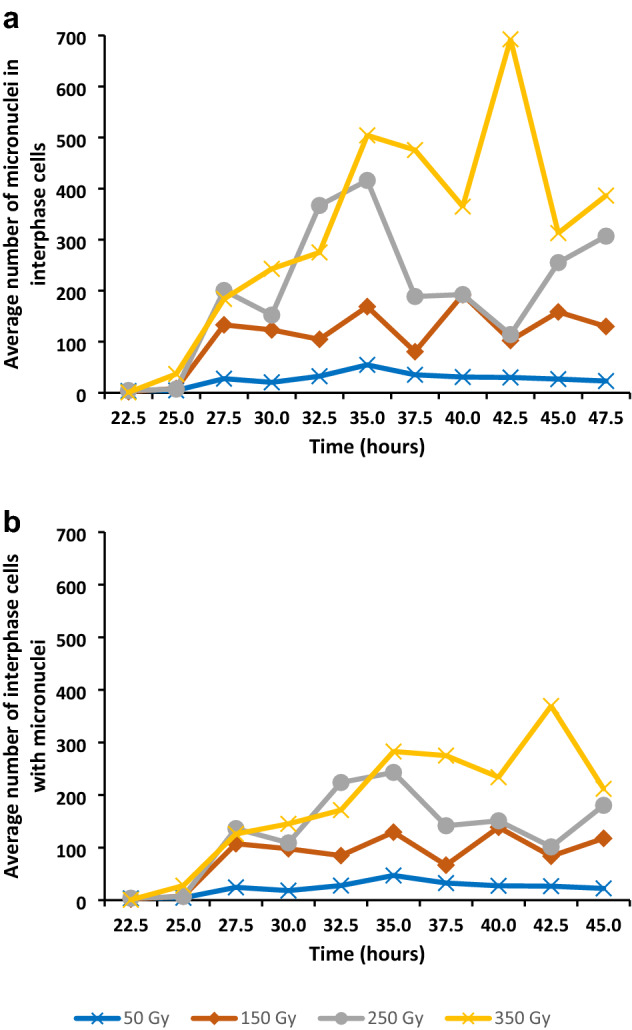
Average number of micronuclei and average number of cells containing micronuclei in *T. turgidum* ssp. *durum* L. cv. Orania. **a** Average number of micronuclei in 500 interphase cells. **b** Average number of interphase cells containing micronuclei per 500 cells

The total number of micronuclei at various times after the onset of imbibition was determined to obtain an understanding of the effect of gamma irradiation at different doses (Fig. 8a). The data revealed that there was an increase in the number of micronuclei with an increase in dose, with a mean number of about 24 micronuclei in 50 Gy material to 310 micronuclei in 350 Gy material.

The total number of micronuclei by means of the ANOVA tests showed highly significant effects of dose, time and dose by time interaction (Table 4).

The effect of gamma irradiation on the total number of micronuclei according to the LSMEANS procedure revealed highly significant differences among the treatment doses (Table 5.).

The effect of gamma irradiation on the number of micronuclei was also supported by an increase in the total number of cells containing micronuclei (Fig. 8b) with an increase in gamma irradiation dose. When the pattern of the total number of micronuclei and the total number of cells containing micronuclei over time were compared, it was found that the developmental patterns were very similar.

### Incomplete mitosis

**Fig. 9:**
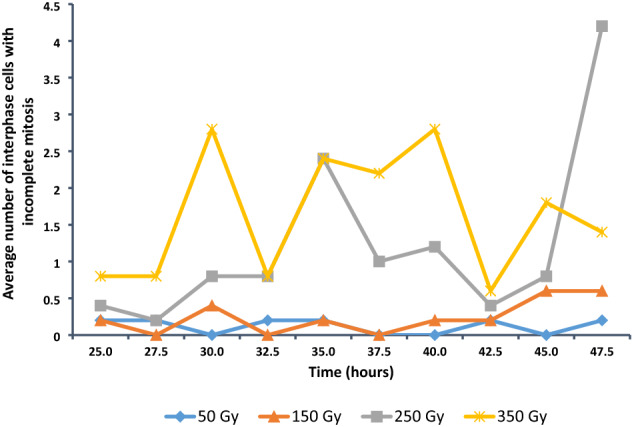
Average number of interphase cells with incomplete mitosis out of 500 interphase cells in *T. turgidum* ssp. *durum* L. cv. Orania

In *Triticum turgidum* ssp. *durum* L. the maximum number of nucleoli in the nucleus is four, formed by NOR’s on chromosomes 1B and 6B (Hutchinson and Miller 1982; Carvalho et al. 2010). Incomplete mitosis was scored as cells with five (Fig. 2i) and more nucleoli in the nucleus resulting from separation of the chromatids during mitosis which then get packed in the same nucleus during interphase, leading to a nucleus in interphase with twice as much DNA (Von Well et al. 2021). At all the irradiation doses interphase cells with incomplete mitosis were seen from 25 h after the onset of imbibition, with an increase of incomplete mitosis with increasing irradiation dose. Fifty Gy and 150 Gy samples had four and three collection times with no incomplete mitosis interphase cells, respectively, while 250 Gy and 350 Gy samples showed interphase cells with incomplete mitosis at all the collection times from 25 h onwards (Fig. 9).

The first observations of interphase cells with incomplete mitosis were at 25 h; by this time, many mitotic divisions had completed. It is expected that the changes over time would vary and the effects of the different gamma irradiation doses would vary as well. The total number of interphase cells with incomplete mitosis by means of the ANOVA tests showed highly significant effects of dose, time and dose by time interaction (Table 4).

The lower irradiation doses are expected to have little impact on the number of interphase cells with incomplete mitosis, while the higher irradiation doses will have a larger effect. The effect of gamma irradiation on the total number of interphase cells with incomplete mitosis according to the LSMEANS procedure revealed non-significant differences among the control, 50 Gy and 150 Gy. Highly significant differences were obtained between the control and the lower doses on the one side and 250 Gy and 350 Gy on the other side, and also between 250 Gy and 350 Gy irradiation dose (Table 5.).

### Correlations between seedling and root growth and the efficiency of energy conversion on the one side and presence of bridges, micronuclei and interphase cells with incomplete mitosis on the other

Correlations between seedling and root growth and the efficiency of energy conversion into growth on the one side and presence of bridges, micronuclei and interphase cells with incomplete mitosis on the other were determined to evaluate if seedling and root growth are measuring different effects of gamma irradiation than the efficiency of energy conversion into growth does (Table 6).

**Table 6:** Correlations between the effects of gamma irradiation on seedling and root growth and efficiency of energy conversion into growth on the one side, and the frequency of cells with different numbers of bridges, the frequency of cells with different numbers of micronuclei and the frequency of cells with incomplete mitosis on the other in *T. turgidum* ssp. *durum* L. cv. Orania

Bridges	Seedling growth		Root growth		Efficiency of energy conversion into growth	
	*p* value	Pearson’s correlation coefficient	*p* value	Pearson’s correlation coefficient	*p* value	Pearson’s correlation coefficient
1 Bridge per cell	< 0.01	− 0.81	< 0.01	− 0.86	0.06	− 0.44
2 Bridges per cell	< 0.01	− 0.90	< 0.01	− 0.90	< 0.01	− 0.65
3 Bridges per cell	< 0.01	− 0.79	< 0.01	− 0.74	< 0.01	− 0.68
4 Bridges per cell	0.67	− 0.16	0.59	− 0.20	0.89	0.05
5 Bridges per cell	0.33	− 0.43	0.36	− 0.41	0.22	− 0.53

Micronuclei	Seedling growth		Root growth		Efficiency of energy conversion into growth	
	*p* value	Pearson’s correlation coefficient	*p* value	Pearson’s correlation coefficient	*p* value	Pearson’s correlation coefficient
1 Micronucleus per cell	< 0.01	− 0.91	< 0.01	− 0.96	< 0.01	− 0.59
2 Micronuclei per cell	< 0.01	− 0.94	< 0.01	− 0.92	< 0.01	− 0.77
3 Micronuclei per cell	< 0.01	− 0.91	< 0.01	− 0.86	< 0.01	− 0.83
4 Micronuclei per cell	< 0.01	− 0.89	< 0.01	− 0.84	< 0.01	− 0.86
5 Micronuclei per cell	< 0.01	− 0.83	< 0.01	− 0.78	< 0.01	− 0.84
6 Micronuclei per cell	< 0.01	− 0.77	< 0.01	− 0.70	< 0.01	− 0.77
7 Micronuclei per cell	0.03	− 0.43	0.06	− 0.37	< 0.01	− 0.62

Incomplete Mitosis	Seedling growth		Root growth		Efficiency of energy conversion into growth	
	*p* value	Pearson’s correlation coefficient	*p* value	Pearson’s correlation coefficient	*p* value	Pearson’s correlation coefficient
Interphase cells with incomplete mitosis	< 0.01	− 0.87	< 0.01	− 0.85	< 0.01	− 0.84

The relatedness between seedling and root growth with the presence of bridges (Table 6) displayed highly significant correlations between seedling and root growth with the presence of one to three bridges per cell with the highest correlation with two bridges per cell. The efficiency of energy conversion on the other hand displayed highly significant correlations with two to three bridges per cell with the highest correlation with three bridges per cell.

The relatedness between seedling and root growth with the presence of micronuclei (Table 6) displayed highly significant correlations with one to six micronuclei per cell. Seedling growth had the highest correlation with two micronuclei and root growth with one micronucleus. The efficiency of energy conversion into growth on the other hand had highly significant correlations with one to seven micronuclei, with the best correlation with four micronuclei per cell.

The relatedness between seedling and root growth with the presence of incomplete mitosis (Table 6) displayed highly significant correlations with interphase cells with incomplete mitosis. Seedling growth had the highest correlation with interphase cells with incomplete mitosis, followed closely by root growth. The efficiency of energy conversion into growth also had a highly significant correlation with interphase cells with incomplete mitosis and the correlation was only slightly less than root growth.

### Determination of the optimum dose for mutation breeding

Due to the differences in the correlations between root growth, seedling growth and efficiency of energy conversion on the one hand with bridges, micronuclei and interphase cells with incomplete mitosis on the other, it was concluded that the efficiency of energy conversion is measuring other aspects of growth retardation.

In *Triticum monococcum* (Von Well et al. 2021) the 100 Gy and higher gamma irradiation treatments were not entangled with the control like 250 Gy and 350 Gy gamma irradiation treatments in the present study. The 100 Gy treatment differed from the control with *p* = 0.01 in *Triticum monococcum* (Von Well et al. 2021). The 100 Gy determination for the optimal dose for mutation breeding is in line with the suggested doses (100–200 Gy) for practical mutation breeding in *Triticum monococcum* L. by the FAO/IAEA. In the present study 250 Gy differed from the control with *p* = 0.02. This makes it ideal for using *p* = 0.01 for the determination of the ideal dose for mutation breeding.

**Fig. 10:**
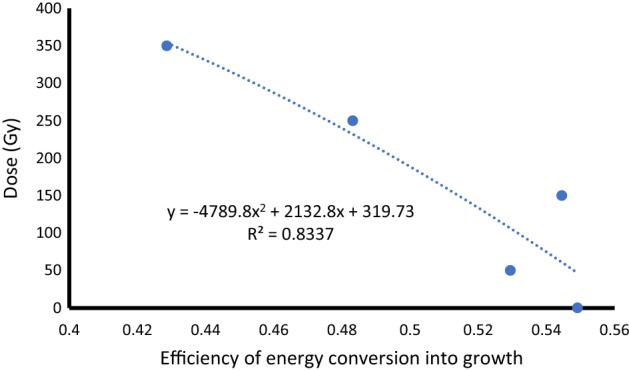
Determination of the ideal gamma irradiation dose for mutation breeding in *T. turgidum* ssp. *durum* L. cv. Orania

The following steps were taken for the determination of the ideal dose for mutation breeding by making use of the efficiency of energy conversion into growth at a *p* value of 0.01. First, the LSD value at *p* = 0.01 was determined as 0.0369. After that, the LSD value was subtracted from 0.54911 to obtain the *x*-axis value of the graph. The x-axis value was determined as: 0.54911–0.0369 = 0.51221. Then, to obtain a best-fit equation the efficiency of energy conversion into growth values was plotted on the x-axis and the corresponding gamma irradiation dose values on the *y-*axis. A quadratic equation, *y* = − 4789.8*x*^2^ + 2132.8*x* + 319.73, resulted in the best fit with *R*^*2*^ = 0.8337 (Fig. 10). Finally, by making use of the equation the ideal dose for mutation breeding was determined as 155.52 Gy.

## Discussion

### Comparisons of the effect of low doses of gamma irradiation on growth parameters

Hormesis is the concept that biological systems can respond in a positive way, or be stimulated by, physical or biologic exposure to low doses of an agent that is toxic at higher doses. Additionally, hormesis is defined as “any physiological effect that occurs at low doses which cannot be anticipated by extrapolating from toxic effects noted at high doses”. Radiation hormesis is the theory that biological systems can respond positively to exposure of low doses of ionizing irradiation (Baldwin and Grantham 2015). Adaptive protection that follows low dose irradiation causes DNA damage prevention, repair, and immune stimulation in animals (Feinendegen 2005). In the present study an increase in shoot growth was observed after 50 Gy gamma irradiation. Similarly, an increase in mitotic index in root apical meristem was observed after 50 Gy gamma irradiation in *Triticum turgidum* ssp. *durum* L. (Von Well et al. 2021). Similar observations were made in hexaploid wheat where lower gamma irradiation doses stimulate, while higher doses inhibit plant growth and development (Irfaq and Nawab 2001; Grover and Khan 2014). All the cultivars responded differently to different gamma ray doses with respect to some of the characters.

### The difference between the response of diploid and polyploid wheat species concerning gamma irradiation

The fact that after 50 Gy gamma irradiation stimulation in shoot growth of *Triticum turgidum* ssp. *durum* L. took place, while retardation in growth took place in *Triticum monococcum* L. means that specific doses would be classified as “low dose irradiation”, depending on and relative to what is being irradiated (Eroglu et al. 2007). In general, the polyploid species are more resistant than the diploid species due to the accumulation of genomes, also known as genetic redundancy, and the combination of different genomes with differences in their irradiation resistance (Fuji and Matsumura 1959; Sree Rangsamy and Sree Ramulu 1970; Ukai 1981).

### Comparisons of the effect of high doses of gamma irradiation on growth parameters

Gamma irradiation had the largest effect on root growth, followed by shoot growth and finally the efficiency of energy conversion into growth. In root growth, all the irradiation doses led to highly significant difference from the control, while samples treated at 250 Gy and 350 Gy differed highly significantly from the controls in shoot growth and the efficiency of energy conversion into growth. Large differences in shoot and root lengths among Basmati rice cultivars were observed after gamma irradiation of the seeds (Ashraf et al. 2003). Root growth was also found to be more sensitive than shoot growth in gamma irradiation of onion seed (Amjad and Anjum 2002). The decrease in growth after high gamma irradiation doses can be attributed to (1) reduced mitotic activity, partly caused by radiation-induced senescence (a condition of permanent cell cycle arrest induced by irradiation) in meristematic tissues and (2) mitotic catastrophe (when cell death occurs during or because of an aberrant mitosis). Senescent cells do not divide but may remain metabolically active. Aberrant mitosis produces an atypical chromosome segregation and cell division and leads to the formation of cells with aberrant nuclear morphology, multiple nuclei, and/or several micronuclei. Giant cells form when cytokinesis does not take place and the cells die over a period. Mitotic catastrophe can occur over a few days (Roninson et al. 2001; Vakifahmetoglu et al. 2008; Eriksson and Stigbrand 2010; Liu et al. 2013; Grover and Khan 2014).

### The presence of bridges, micronuclei and ring chromosomes because of chromatid and chromosome breaks due to gamma irradiation

Gamma irradiation causes chromatid and chromosome breaks which through joining of sticky chromosome or chromatid ends result in anaphase bridges when centromeres are included, while acentric fragments become micronuclei at late telophase. These bridges may persist to telophase and break at the end of mitosis (usually towards the end of telophase) where they can be observed as broken bridges. Gamma irradiation of chromatin occurred in cells in G_1_ phase of the cell division cycle, when all chromatin threads are single stranded, as well as cells in S and G_2_ phase, which have replicated through the S phase of the cell division cycle. Ring chromosomes originate by breaks in both chromosome arms. The appearance and increase of ring chromosomes is therefore expected to occur at relatively higher doses. We found that the samples treated with 150 Gy, 250 Gy and 350 Gy irradiation doses show a highly significant increase in bridges and micronuclei in comparison with 50 Gy samples, while only samples treated with 250 Gy and 350 Gy showed a highly significant increase in ring chromosomes in comparison with 50 Gy samples. A highly significant increase in mitotic cells with chromosomal aberrations with increasing irradiation dose was also observed in eight winter wheat cultivars treated with 100, 150, 200 and 250 Gy. The more susceptible cultivars showed a larger reduction in mitotic activity and more chromosomal aberrations (Nazarenko and Kharytonov 2016). Oney-Birol and Balkan (2019) also observed a decrease in mitotic index in two out of three bread wheat cultivars over a dose range of 100–300 Gy gamma irradiation. Azer (2001) concluded that irradiation with less than 100 Gy was needed to induce 50% abnormal cells in two bread wheat cultivars, while a dose between 300 Gy and 400 Gy were needed to induce the same effect in another cultivar.

### The relationship between the efficiency of energy conversion into growth and the presence of bridges, ring chromosomes and micronuclei

Physiological processes, as well as cell death and cellular senescence influence root, shoot and seedling growth, while the efficiency of energy conversion into growth is mainly influenced by cell death, cellular senescence and cellular repair efficiency. Doses which, on average, led to one or two bridges or micronuclei per cell had the largest effect on root, shoot and seedling growth. This can be attributed to altered physiological processes such as decreased NOR activity (Von Well et al. 2021) or G_2_ cell cycle arrest for DNA repair at an early checkpoint and a later checkpoint (Xu et al. 2002; O’Connell and Cimprich 2005; Zhao et al. 2019). The efficiency of energy conversion into growth correlates best with on average three bridges and four micronuclei per cell, as mainly occur after 250 Gy gamma irradiation. This is the irradiation dose leading to a highly significant increase in incomplete mitosis in comparison with 50 Gy. Higher correlations were obtained with root, shoot and seedling growth and the efficiency of energy conversion into growth with micronuclei per cell than with bridges per cell. Silva-Barbosa et al. (2005) also concluded that it is easier to make use of micronuclei than bridges (dicentrics) when studying dose dependence.

Incomplete mitotic division, which may be the result of a combination of chromosomal abnormalities, was seen in all the irradiation doses and increased with dose (Gupta et al. 2019). Incomplete mitosis is detected by the occurrence of five to six nucleoli per nucleus and seven to eleven nucleoli per cell, as well as a decrease in the frequency of cells in telophase (Al-Safadi and Simon 1990; Von Well et al. 2021). These cells die over a longer period, which, together with cellular senescence, is responsible for the decrease in the efficiency of energy conversion into growth. Seedling growth had the highest correlation with incomplete mitosis. The efficiency of energy conversion into growth is estimated by seedling growth divided by the energy used to obtain the specific growth. It can therefore be concluded that the energy used for the repair of damaged cells that are able to divide outweighs the energy lost due to incomplete mitosis. Avanzi and Deri (1969) determined the durations of the mitotic cycle in two *Triticum turgidum* ssp. *durum* L. cultivars as 14 h for Aziziah and 14 h and 15 min for Capelli. Kaltsikes (1971) measured a mitotic cell cycle duration of 13 h and 45 min in a *Triticum turgidum* ssp. *durum* L. cultivar. According to the mitotic activity of the control and 50 Gy, they have started with a second mitotic cycle (Von Well et al. 2021). At the end of the 132 h for the determination of the efficiency of energy conversion into growth they would have completed 6–7 mitotic cycles, while samples treated with 150 Gy, 250 Gy and 350 Gy would have completed 4–6 cycles. These cycles are enough for death by mitotic catastrophe or apoptosis to take place, as seen in the deterioration of the efficiency of energy conversion into growth observed in 250 Gy and 350 Gy samples over time.

### Finding the optimal dose for mutation breeding.

In the present study, response curves after 250 Gy and 350 Gy gamma irradiation were not entangled with the control like the 100 Gy samples in *Triticum monococcum* L. (Von Well et al. 2017). This is due to a highly significant increase in incomplete mitosis between 150 and 250 Gy in *Triticum turgidum* ssp. *durum* L which supports the prediction of an optimal gamma irradiation dose between 150 and 250 Gy. According to FAO/IAEA (FAO/IAEA 2018), the ideal gamma irradiation dose range for mutation breeding in *Triticum turgidum* ssp. *durum* L. is 150–300 Gy. The ideal gamma irradiation dose of 155.52 Gy falls in this range. We propose to use the efficiency of energy conversion into growth as an ideal method for the prediction of the ideal dose for mutation breeding.

## Data Availability

No sequencing data was generated by the study. The datasets used and/or analysed during the current study are available from the corresponding author on reasonable request.
